# Structured ternary fluids as nanocrystal incubators for enhanced crystallization control†

**DOI:** 10.1039/d2sc04413g

**Published:** 2022-10-24

**Authors:** J. J. Maunder, J. A. Aguilar, P. Hodgkinson, S. J. Cooper

**Affiliations:** Department of Chemistry, University of Durham Durham DH1 3LE UK sharon.cooper@durham.ac.uk

## Abstract

In crystallization from solution, a ubiquitous process in both industry and the natural world, nucleation is usually the rate-determining step, followed by faster crystal growth. Consequently, crystals typically exist in the nm-size range for such limited times that their investigation and manipulation is hindered. Here, we show that, owing to a degree of restricted diffusion, crystallization in structured ternary fluids (STFs) can proceed *via* higher nucleation rate and slower crystal growth pathways. This enables STFs to act as nanocrystal incubators, with the nanocrystals existing for extended times. We demonstrate that this generates enhanced crystallization control, with the three ambient pressure polymorphs of glycine, the α-, γ- and β-forms, all crystallizing from the octanol/ethanol/water STF, despite the well-known difficulty in crystallizing the slow growing γ-form and the instability of the β-form. The ability of STFs to produce notoriously hard to crystallize polymorphs should make them a versatile tool, ideal for polymorph discovery. This may enable a step change in the current, scatter-gun approach to polymorph screening. Furthermore, we show that aliquots of the nanocrystal-containing fluids can successfully seed metastable solutions. Hence, STFs may ultimately help provide a generic methodology for producing crystals and seed suspensions of any desired polymorph to supersede current targeted crystallization and seeding strategies.

## Abstract 2

Crystallization in structured ternary fluids can proceed *via* higher nucleation rate and slower crystal growth pathways that are impossible to access in normal unstructured solutions. Hence, structured ternary fluids can act as nanocrystal incubators.

## Graphical abstract

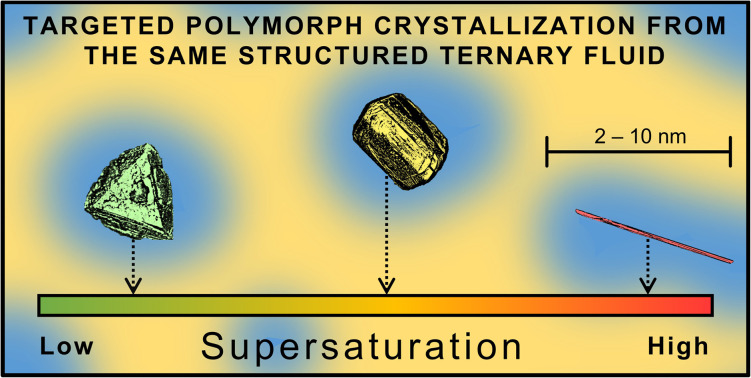

## Introduction

Crystallization is ubiquitous in both nature and industry; the formation of nanomaterials, ceramics, pharmaceuticals, biominerals and rocks all rely on this process. Despite this prevalence, crystallization mechanisms remain poorly understood and controversial.^1^ Here we introduce crystallization studies in a class of materials, structured ternary fluids (STFs), to help address these fundamental issues.

STFs consist of two immiscible liquids, typically an oil and water, and an amphi-solvent that is miscible with both liquids. Although they were first reported in 1977,^2^ little research was conducted on these systems until recently,^3–13^ when the presence of dynamic nano-sized domains of aqueous and oil regions was convincingly shown *via* small angle X-ray and neutron scattering,^3–7^ and static and dynamic light scattering.^8^ NMR, conductivity, UV-vis spectrometry of probe molecules and molecular dynamics have provided additional evidence to support the existence of STFs.^9–13^ The presence of the ≈2–10 nm-sized domains means that these STFs are mimics of surfactant-based microemulsions. Consequently, STFs are also known as surfactant-free microemulsions and ultraflexible microemulsions, with the latter emphasizing the more flexible, longer-range and less distinct interface between the oil and aqueous regions, which means the nanostructuring, though always present, is more dynamic and less defined.

To-date, there have been only a few studies on particle formation in STFs. These studies involved inorganic or metal nanoparticles, and focused on the particle morphologies obtained, rather than the crystallization kinetics.^14–19^ Consequently, the effect of STF nanoconfinement on crystallization has not been considered. In this work, we reveal the unique crystallization kinetics in these systems and demonstrate the extensive potential these systems have for understanding and controlling crystallization.

**Fig. 1:**
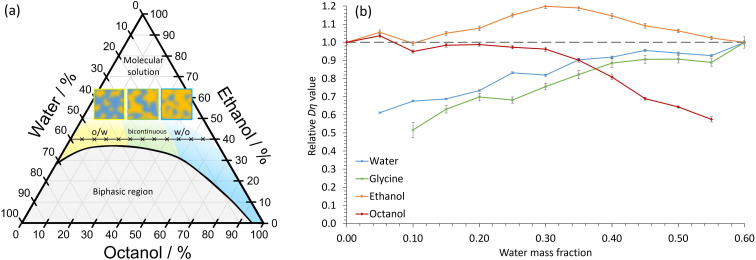
Nanoconfinement in the octanol/ethanol/water ternary system. (a) Phase diagram by percentage mass. o/w and w/o refer to oil-in-water and water-in-oil nanostructures, respectively. The compositions of the STFs investigated, which contain 40 wt% ethanol, and varying amounts of water and ethanol, are shown by crosses. The schematic diagrams depict o/w, bicontinuous and w/o STFs. (b) The product *Dη*·relative to the *Dη*·value in the corresponding binary system (*i.e.* water/ethanol for water and glycine, and octanol/ethanol for octanol), as a function of water mass fraction. For ethanol, the *Dη*·product is relative to the weighted average of the water/ethanol and octanol/ethanol binary systems. The error bars show the standard error in *Dη*·from 3 repeats for each diffusion and viscosity experiment.

In our experiments, the immiscible liquids octanol and water were mixed in combination with the amphi-solvent, ethanol. The octanol/ethanol/water STF has been well studied,^3–8,11^ with the nanostructuring occurring close to the two-phase boundary (Fig. 1a). At low water volume fractions, dynamic hydrogen-bonded hydroxyl networks of nm-size occur, which swell as the water content increases, whereas nm-sized globular pockets of octanol are present at high water fractions.^7^ For similar oil and water volume fractions, more bicontinuous structures form. The ethanol is proportioned approximately equally between the aqueous and octanol domains, with a slight excess in the interfacial regions.^7^ The lifetime of the nanodomains in STFs is not currently known. However, the structures must exist for times greater than the 10–100 ns correlation times of dynamic light scattering experiments.^8^ Consequently, we hypothesized that the nanoconfined regions may be sufficiently long-lived to radically impact crystallization and provide greater polymorph control. This contrasts with the ≈1 nm clusters in binary water–alcohol systems that have lifetimes of <50 ps (ref. 20) – too short to impart noticeable nanoconfinement effects on crystallization.

The problem of crystallizing a desired polymorph in many systems is well established.^21,22^ In the case of glycine, it is difficult to crystallize the stable, but slow-growing, γ-form from aqueous solutions.^23–25^ Instead, the metastable α-polymorph typically crystallizes.^26^ The metastable β-form can crystallize by rapidly adding the antisolvent ethanol to aqueous glycine solutions, thereby achieving high supersaturations so as to nucleate the β-form, but solution-mediated transformation to the more stable α-polymorph is rapid, particularly for solutions with high water content.^27,28^ Therefore, glycine was an ideal candidate to test the effect of STFs on crystallization. Here we reveal that using an STF dramatically alters the crystallization outcome compared to unstructured solutions.

The rate-determining step in crystallization from solution is the initial nucleation process, owing to its larger energy barrier, with crystal growth proceeding more quickly. We show here, for the first time, that crystallization can proceed in STFs *via* higher nucleation rate and slower crystal growth regimes that are impossible to achieve in normal unstructured solutions. This enables enhanced control over the crystallization process such that all three polymorphs of glycine can be produced from the same STF. The origins of this higher nucleation rate/slower growth mechanism lie in the soft nanoconfinement of the glycine, characterized by the extent of its restricted diffusion. Furthermore, the higher nucleation rate/slower growth mechanism means nanocrystals can exist for extended times in the STF. We exploit this and show that aliquots of the STF can successfully seed metastable solutions. We hope that the significant advantages that crystallization in STFs provide over current targeted crystallization and seeding strategies lead to their adoption throughout the crystallization field.

## Results and discussion

To investigate the extent of the glycine nanoconfinement, we measured the apparent diffusion coefficient, *D*, of each component in different STF mixtures using NMR diffusiometry. When molecules rebound from boundaries, as in nanoconfined systems, the measured diffusion coefficient is smaller than its value in an unrestricted solution because the apparent travel distance is shorter. Hence NMR diffusiometry provides a direct probe of the degree of confinement of the different components.

The diffusion coefficient of a component in a liquid mixture with viscosity, *η*, is given by the Stokes–Einstein equation,
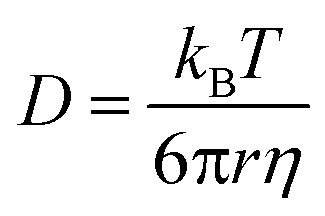
where *k*_B_ is Boltzmann's constant, *T* is temperature and *r*, the Stokes radius, is the radius of a hard sphere that diffuses at the same rate as the component particle. The Stokes radius for a given component will depend upon the surrounding solution, but for formulations containing the same constituents at the same temperature, *Dη* is approximately constant for a particular component in the absence of nanoconfinement. In nanoconfined systems, *Dη* will decrease because of the smaller apparent diffusion coefficient. To facilitate comparison between the different STFs, *Dη* values are plotted in Fig. 1b relative to the corresponding binary mixture, with values significantly decreased from 1 showing greater levels of nanoconfinement. *D*, *η* and absolute *Dη* values are shown in Fig. S1.†

From Fig. 1a, we expect water to be the confined phase in mixtures with low water mass fraction. Therefore, the apparent *Dη* should decrease as the water content reduces. This is what we observe (Fig. 1b); there is a ≈40% decrease in the apparent *Dη* for water at low water mass fractions relative to the 0.60 water/0.40 ethanol mass fraction binary system, consistent with soft nanoconfinement. Even at water mass fractions of 0.25, the values are reduced by ≈20% compared to the binary system. We expect octanol to be confined in STFs with high water content and, again, this is what we observe. Ethanol, on the other hand, remains unconfined at all compositions. We note here that although ethanol's *Dη* value increases slightly in the bicontinuous region, it never decreases significantly, indicating that there is no restriction. The magnitude of these *Dη* changes are similar to those reported in a STF composed of an ionic liquid, ethanol and toluene.^9^ Thus, in agreement with previous studies,^3–8,11^ these findings confirm that soft nanoconfinement is present in the ternary 0.40 mass fraction ethanol systems studied.

Note that Fig. 1a shows that nanoconfinement is lost as ethanol content increases in the ternary mixtures. For ternary fluids with a greater ethanol mass fraction of 0.60, the relative *Dη* values reflect this as they remain similar for all components in all compositions (Fig. S2†).

Crucially, Fig. 1b shows that the relative *Dη* values for glycine closely follow those of the water component, the only difference being slightly lowered relative *Dη* values at low water content. This is consistent with glycine diffusion being restricted because the glycine resides primarily within dynamic water pockets and is largely excluded from the interfacial regions, as expected due to glycine's poor solubility in both ethanol and octanol (Fig. S3 and Table S1†).

Nanoporous materials and droplet microemulsions provide considerably greater restricted diffusion compared to STFs.^29,30^ For instance, in droplet microemulsions, diffusion coefficients can be reduced by 1–2 orders of magnitude.^30^ Instead, the degree of restricted diffusion here is more similar to that of oils and aqueous phases in bicontinuous surfactant-based microemulsions.^31^ Nevertheless, this degree of restricted diffusion is sufficient to cause transformative effects on the crystallization behaviour, as we show below.

### Crystallization of γ-glycine

Crystallization experiments were conducted on the 0.40 ethanol mass fraction octanol/ethanol/water formulations, since these are close to the 2-phase boundary and hence show nanostructuring, as demonstrated above. The glycine polymorph was principally determined by ATR-FTIR, with characteristic peaks for α, β and γ-glycine occurring at ≈909, 914 and 928 cm^−1^, respectively.^27,32,33^ Powder X-ray diffraction data on selected samples fully corroborated the ATR-FTIR results (Fig. S4†).

We first used slow cooling to induce γ-glycine crystallization. This will typically favour thermodynamically stable polymorphs because the system spends sufficient time at higher temperatures where only the stable polymorph is supersaturated. This strategy fails for γ-glycine in aqueous solution, however, because the metastable α-polymorph is only slightly less stable than the stable form,^23^ and γ-glycine has a much lower growth rate.^23,24^ Consequently, nanocrystals of α-glycine can grow to larger dimensions more rapidly, ultimately becoming more stable than the much smaller γ-glycine nanocrystals, which then dissolve whilst the α-glycine crystals continue to grow. Therefore, to selectively crystallize γ-glycine, specific additives, or a pH away from the isoelectric point, are required.^34–36^

**Fig. 2:**
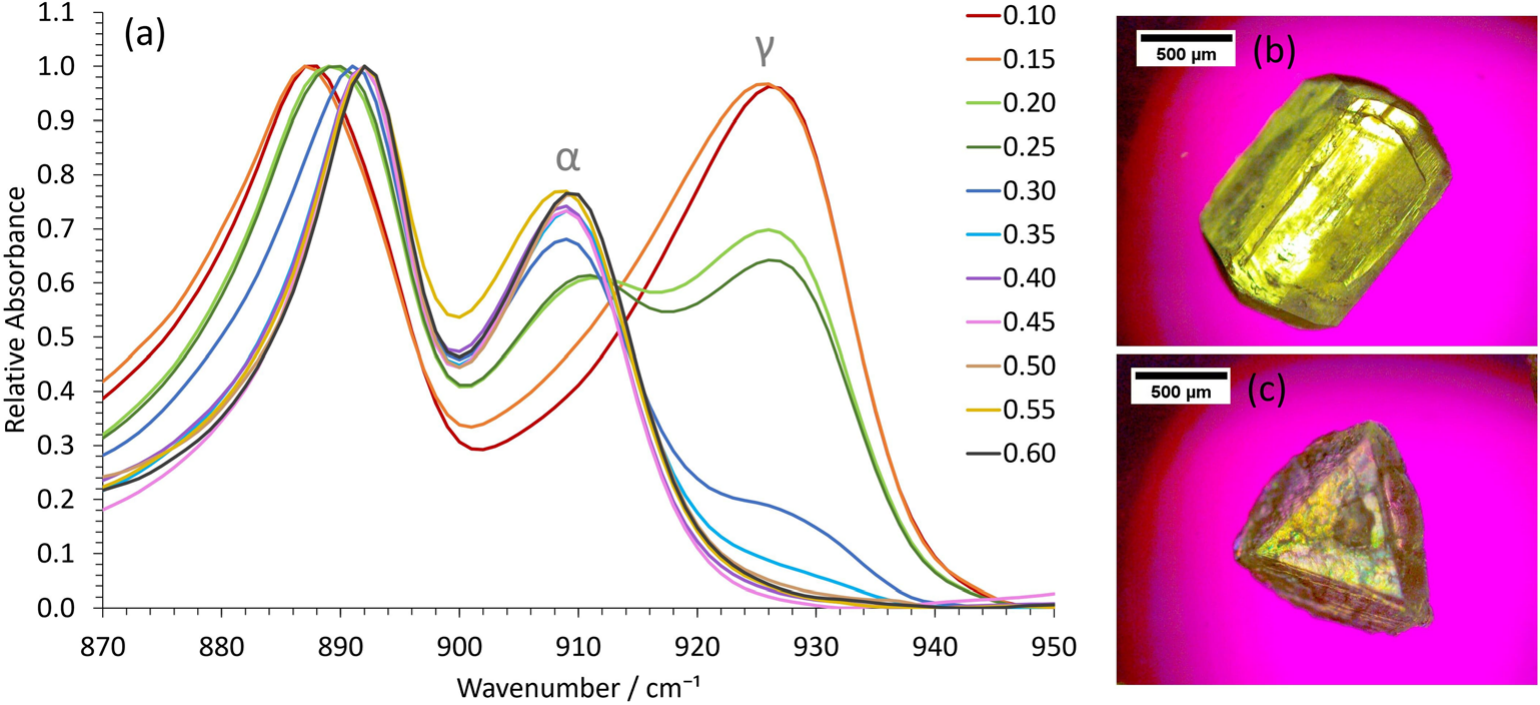
Obtaining γ-glycine in low water mass fractions STFs at low supersaturations with *c*/*c*_sat_ = 1.30. (a) Representative ATR-FTIR spectra of extracted crystals as a function of water mass fraction in the STF. The spectra have been normalized relative to the common peak at 890 cm^−1^. Optical micrographs showing the typical morphology of (b) α-glycine crystals obtained from the binary system and higher water mass fraction STFs, and (c) γ-glycine crystals obtained from the lower water mass fraction STFs.

In our water-in-oil STF compositions containing 0.10 mass fraction of water, γ-glycine was successfully crystallized as the sole product by using slow cooling for supersaturations with *c*/*c*_sat_ = 1.30 (Fig. 2), where *c*_sat_ and *c* are the saturation and actual concentrations, respectively; this was also confirmed by powder X-ray diffraction (Fig. S4†). For water-in-oil STF compositions of 0.15 mass fraction of water, γ-glycine crystallized as the majority polymorph, with only a minor amount of α-glycine present. In bicontinuous STF compositions of 0.20 and 0.25 mass fraction of water, both α- and γ-glycine typically crystallized concomitantly, with γ-glycine usually being the predominant polymorph at supersaturations with *c*/*c*_sat_ = 1.30. At higher water mass fractions, where octanol replaces water as the nanoconfined phase to give oil-in-water structures (Fig. 1a), the ability to crystallize γ-glycine was markedly reduced, as expected. In particular, in formulations containing 0.30 and 0.35 mass fraction of water, α-glycine was the majority polymorph, with some γ-glycine still evident, whilst for mass fractions of water ≥ 0.40, only α-glycine was evident at *c*/*c*_sat_ values of 1.30. Note that a faster-cooling method was also able to crystallize γ-glycine, provided STFs with water mass fractions of ≤0.25 were used at *c*/*c*_sat_ values of 1.30 (Fig. S5†). This is consistent with the nanocrystals growing more independently from one another in the same STF due to the restricted diffusion, enabling smaller, less stable nanocrystals to persist. In contrast, when octanol/ethanol/water unstructured solutions containing 0.60 mass fraction of ethanol were used, only α-glycine crystallized (Fig. S6†).

### Crystallization of β-glycine

Whilst the stable γ-glycine polymorph could be crystallized in our STFs by using low supersaturations, the metastable β-glycine polymorph could be targeted at higher supersaturations. We focused on the 0.35 octanol/0.40 ethanol/0.25 water mass fraction formulation since, although in the bicontinuous region, it was sufficiently close to the water-in-oil boundary for 3D nanoconfinement effects to be apparent, as demonstrated by its ability to crystallize γ-glycine. This formulation was preferable to lower water mass fraction water-in-oil STFs, even though these possessed greater 3D nanoconfinement, because of the significantly greater glycine amounts that could be dissolved (Table S1†), and the ease with which we could tailor the system to produce all three ambient pressure polymorphs of glycine.

**Fig. 3:**
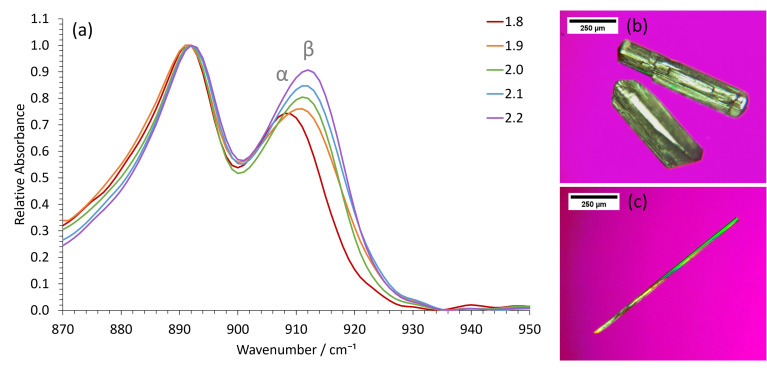
Obtaining β-glycine in the 0.25 water mass fractions STFs at higher supersaturations with *c*/*c*_sat_ ≥ 1.90. (a) Representative ATR-FTIR spectra of extracted crystals as a function of *c*/*c*_sat_. The spectra have been normalized relative to the common peak at 890 cm^−1^. Optical micrographs showing the typical morphology of (b) α-glycine crystals obtained from the binary system and higher water mass fraction STFs and (c) β-glycine crystals obtained from the 0.25 water mass fraction STFs.

β-Glycine crystals could be extracted alongside the more stable α-glycine from STFs with 0.25 mass fraction of water at supersaturations with *c*/*c*_sat_ of ≥1.90 for up to 3.5 hours (Fig. 3). Here, the ability of nanocrystals within the same STF to exist more independently of one another is beneficial. In particular, the restricted diffusion in STFs hinders crystal growth and Ostwald ripening, allowing less stable forms that nucleate to survive alongside more stable, faster growing polymorphs. Furthermore, the restricted diffusion of the nanoconfined immiscible liquid and its solute means that any locally high supersaturations will be prolonged, facilitating the nucleation of polymorphs that would normally be difficult to nucleate.

For instance, assuming a Poisson distribution of solute molecules amongst aqueous swollen pockets of mean size 4 nm, then for a supersaturation with *c*/*c*_sat_ of 2, a 100 ml 0.25 water mass fraction STF would have ≈0.1% of aqueous pockets with supersaturations with *c*/*c*_sat_ in excess of 4, *i.e.* ≈10^16^ pockets that could act as sites for nucleation of high energy polymorphs. Consequently, even if a metastable polymorph were significantly more soluble than the stable form, locally high supersaturations sufficient for nucleation of this highly metastable form should be present within the STF. In solutions lacking this aqueous nanoconfinement, such locally high supersaturations would be transitory because they would be rapidly dissipated by the osmotic pressure arising from the concentration gradient. Hence, only α-glycine crystallized from the binary 0.60 water/0.40 ethanol mass fraction solution at this supersaturation level. Indeed, significantly higher supersaturations with *c*/*c*_sat_ of ≥2.4 were required before β-glycine nucleated concomitantly with α-glycine in this binary system, and this β-glycine did not persist, as it underwent a solution-mediated phase transformation to α-glycine within 30 minutes (Fig. S7†).

We could also obtain virtually 100% β-glycine, as verified by powder X-ray diffraction (Fig. S4†), by simply scaling up the STF with 0.25 mass fraction of water and *c*/*c*_sat_ of 2.2 from 25 ml to 100 ml. This, we suspect, is because the hydrophilic glass walls are wetted more by the aqueous phase of the STF, thereby reducing the nanoconfinement in the vicinity of these walls so that local α-glycine crystallization is more likely here. Accordingly, crystallization from larger volumes that have smaller surface area to volume ratios minimizes this container wall effect. The formation of α-glycine crystals adhered to the vertical glass sides supports this hypothesis.

### Crystallization mechanism

The crystallization outcomes for the STFs containing ≤0.25 mass fraction of water are consistent with a mechanism that is markedly altered compared to crystallization in normal, unstructured solutions. If a solute is soluble in only one of the immiscible liquids of an STF, and specifically the minor component one, the solute is largely confined to the nm-sized pools of that liquid and a degree of restricted diffusion arises, as we have shown. Then, as a crystal nucleus grows in a nm-sized pool, there is a significant supersaturation depletion, both from the limited amount of solute in the nm-sized pool, and the restricted diffusion that impedes additional solute molecules entering the pool. This supersaturation depletion hinders the emergence of stable nuclei. Consequently, to achieve crystallization, a higher initial supersaturation is required, which in turn, results in a higher nucleation rate. We hypothesize that the higher nucleation rate will be further enhanced by two additional factors. First, the large amount of interface, albeit diffuse, in the STF can help induce solute ordering. Secondly, the restricted diffusion of the immiscible liquids and the solute means that any locally high supersaturations will be prolonged, further facilitating nucleation.

Although the nucleation rate is higher in the STFs, crystal growth of the nuclei is quickly reduced due to the rapid supersaturation depletion as the nuclei grow in their nanoconfined regions. This reduced crystal growth continues until the nuclei or nanocrystals are surrounded by nm-sized pools of saturated solution. Further growth of the larger, more stable nanocrystals is then expected to occur predominantly *via* Ostwald ripening through the dissolution of smaller, less stable nuclei or nanocrystals. However, this process is severely hampered by the restricted diffusion of the solute. Hence, the crystallization profile in the STFs is distinctly different to crystallization in normal, unstructured solutions. In unstructured solutions, nucleation is usually the slow step, resulting in a limited population of nuclei that then grow rapidly past the nm-size range; in STFs, the higher nucleation rate and slower crystal growth create numerous nuclei that will then grow exceedingly slowly past the nm-size range if the local supersaturation has been depleted. Essentially, the STF can act an as an array of nanocrystal incubators when a suitable initial supersaturation is used.

**Fig. 4:**
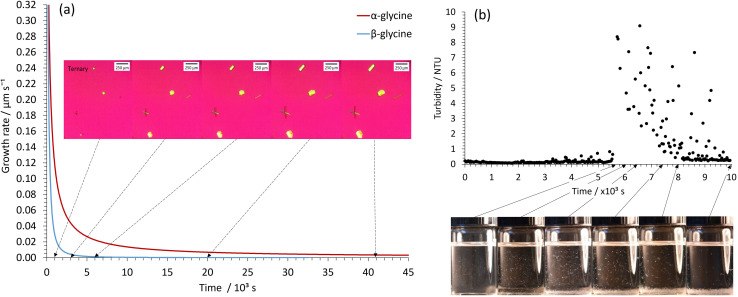
Higher nucleation rate, slower growth mechanism in the 0.25 water mass fraction STF revealed by optical microscopy, visual observation and turbidity measurements. (a) Optical micrographs showing 3 α-glycine crystals and 3 β-glycine needle crystals along with the average growth rate plot for the α- and β-glycine crystals during crystallization in the *c*/*c*_sat_ = 1.90 system. (b) Turbidity measurements and visual observation of suspended crystals in the *c*/*c*_sat_ = 1.90 system. The suspended crystals are particularly evident in the middle vials but have mostly sedimented in the far-right vial.

The higher nucleation rate and slower growth profile for glycine crystallization within our STFs is readily apparent from simple visual observation, optical micrographs and turbidity measurements when supersaturations with *c*/*c*_sat_ of 1.90 are employed in the 0.25 water mass fraction STF (Fig. 4). The optical micrographs showed several α- and β-glycine crystals in the field of view, and whilst these crystals initially grew quite rapidly, their growth rate soon plummeted owing to their local supersaturation being significantly decreased. This is evident from the growth rate plots of the α- and β-glycine shown in Fig. 4a. Note that the β-glycine growth plummets more rapidly, and this is expected, owing to the increased solubility of this less stable form. In the turbidity experiments, elevated Nephelometric Turbidity Unit (NTU) readings interspersed with large spikes were consistent with the presence of suspended glycine crystals of size ∼50–100 μm that grew relatively slowly and then sedimented, causing a large NTU increase as they traversed the laser beam (Fig. 4b).

In contrast, for glycine crystallization at the same 1.90 *c*/*c*_sat_ value in the binary 0.60 water/0.40 ethanol mass fraction system, the samples had to be scanned in the optical microscope to locate the much rarer crystals, which then grew at a faster rate to produce larger crystals that rapidly sedimented; hence the crystals mainly just appeared at the base of the vial (Fig. S8†). These findings confirm our premise that crystallization in the STFs proceeds by a novel higher nucleation rate and slower growth pathway. At the lower *c*/*c*_sat_ value of 1.30, the depletion of local supersaturation occurs in the nm crystal size-range for the 0.25 water mass fraction STF, and hence the STF then acts as an array of nanocrystal incubators, with the slowly growing nanocrystals remaining suspended throughout the fluid for extended periods of 16 hours to over a day. We exploited this capability in our seeding experiments.

### Seeding capabilities

**Fig. 5:**
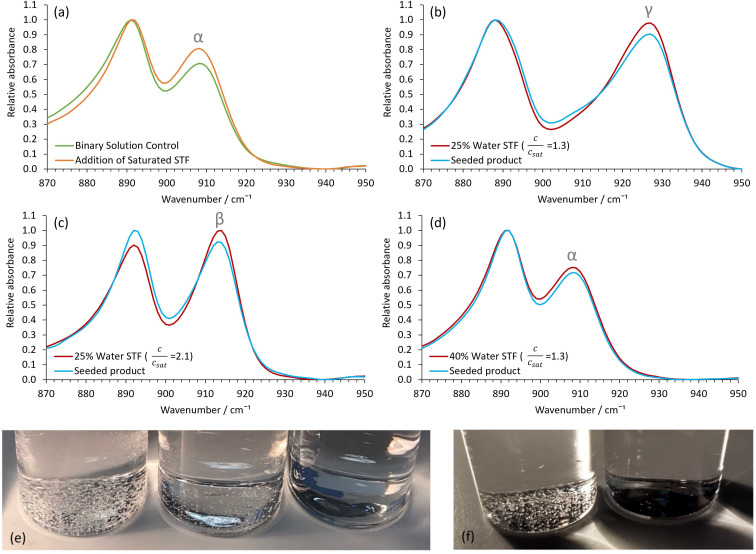
Showing the seeding capabilities of the nanocrystal containing 0.25 water mass fraction STFs. (a) Representative control ATR-FTIR spectra of extracted crystals after eventual ≈ 24-hour α-glycine crystallization in the *c*/*c*_sat_ = 1.30 binary 0.40 water/0.60 ethanol mass fraction system and α-glycine crystallization after ≈ 2 hours when this system had a glycine-saturated STF aliquot added. Representative ATR-FTIR spectra showing (b) γ-glycine crystallization, (c) β-glycine crystallization and (d) α-glycine crystallization in the binary system after seeding with aliquots of the STFs containing seeds of these polymorphs. The ATR-FTIR spectra from the same STFs once the glycine crystals had grown and sedimented are also shown. (e) Photographs taken 24 hours after seeding with the α-glycine (left) and γ-glycine (middle) nanocrystal-containing STFs compared to the control unseeded system (right). (f) Photographs taken 24 hours after seeding with the β-glycine nanocrystal-containing STF (right) compared to the control unseeded system (left).

Aliquots of the nanocrystal-containing STFs were used to seed metastable^37^ supersaturated glycine solutions to induce crystallization of the desired polymorph. The metastable binary solutions contained 0.40 water and 0.60 ethanol mass fractions and had a relative glycine concentration, *c*/*c*_sat_, of 1.30. If left undisturbed, these metastable binary solutions eventually produced α-glycine after ≈1 day. In agreement with previous studies^28,34,38^ γ- and β-glycine crystallization was never observed. Despite this, seeding these metastable binary solutions with aliquots of the γ-glycine nanocrystal-containing STFs resulted in visible γ-glycine crystals forming within a shortened timeframe of 1–2 hours (Fig. S9a†). Similarly, seeding with higher glycine concentration STFs containing β-glycine nanocrystals produced β-glycine in the metastable binary solutions within 10 minutes, whilst seeding with STFs containing α-glycine nanocrystals produced α-glycine, again within a shortened timeframe of 1–2 hours (Fig. S9b and S9c†). Consequently, there is an excellent correlation between the seed nanocrystal polymorphs and the macroscopic crystals produced in the metastable binary solutions (Fig. 5).

Importantly, this suspended nanocrystal seeding method offers distinct advantages over traditional methods that frequently use grinding of macroscopic crystals to produce the seeds. Grinding is an energy-intensive process that often produces high energy defects on the seeds' surfaces, which can then act as sites for secondary nucleation of unwanted polymorphs. In contrast, the isolated suspended nanocrystals grow slowly under the restricted diffusion conditions in the STFs to produce well-formed single crystals that are bounded by low energy faces, even under high supersaturation conditions. This differs from crystallization in unstructured fluids, where attempts to produce nanocrystals from a soluble component by inducing a high supersaturation through crash cooling invariably produce poorly crystalline colloidal particles that rapidly aggregate. Of course, seed suspensions in unstructured solutions can be obtained by antisolvent addition, reactive crystallization, or the use of *e.g.* ultrasound or lasers.^39^ However, these methods do not provide a generic capability for producing longer-lived nanocrystal suspensions of any desired polymorph. STFs may provide this generic capability.

### Polymorph screening capabilities

**Fig. 6:**
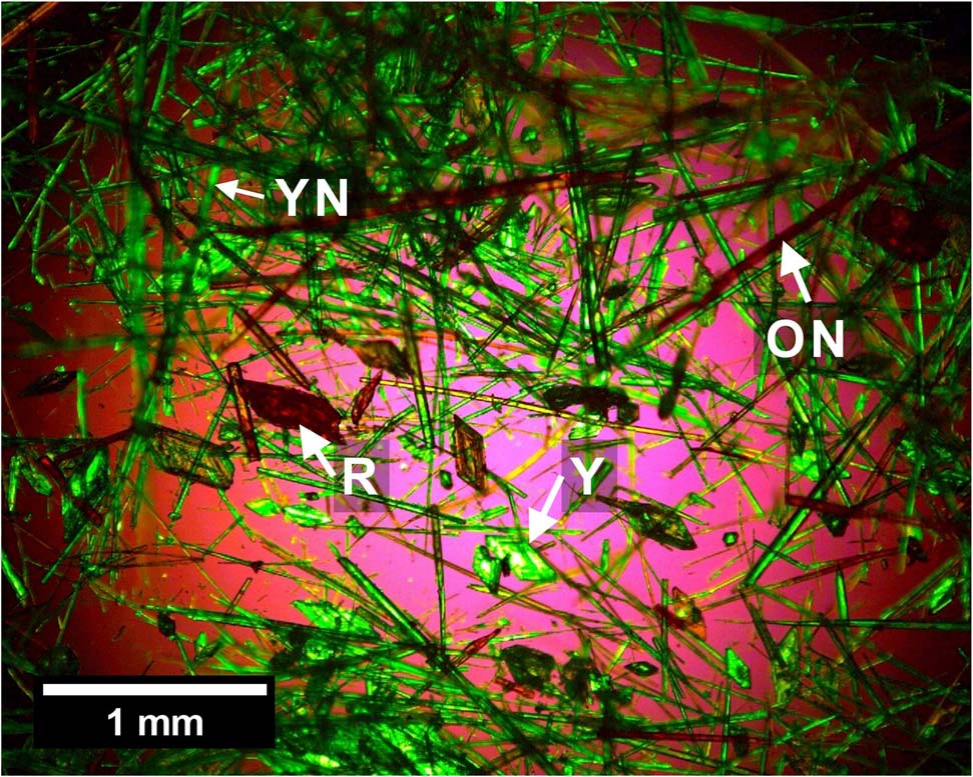
Optical micrograph showing four polymorphs of ROY (YN, ON, R and Y, with representative forms arrowed) from the polymorph screening trial on the toluene/isopropanol/water STF.

To gauge the likely polymorph screening capability of the STFs, we conducted trial experiments at high supersaturation on the highly polymorphic compound, 5-methyl-2-[(2-nitrophenyl)amino]-3-thiophenecarbonitrile, which is commonly known as ROY because of its Red, Orange and Yellow polymorphs. ROY is extremely soluble in toluene and virtually insoluble in water, so the toluene/isopropanol/water STF^40^ was used for these crystallization studies. We successfully crystallized three metastable polymorphs of ROY, the YN, ON and R forms, and the stable Y polymorph within the same STF consisting of 0.100 toluene, 0.525 isopropyl alcohol and 0.375 water mass fractions (Fig. 6), while only the stable Y form was obtained in the binary 0.475 toluene and 0.525 isopropanol mass fraction system. This is noteworthy because the YN polymorph was not reported until 5 years after the Y, ON and R forms,^41,42^ whereas here it appeared at the first attempt. Subsequent crystallizations within this STF were then able to target a particular metastable form because the YN and ON forms appeared prior to R.

The melt crystallized^43^ ROY polymorphs and the most unstable ROY polymorphs obtained through high throughput methods^43,44^ and the use of specific additives^45^ were not crystallized in this initial STF trial. To induce their STF crystallization, these would likely need, for example, switching to a different STF or inclusion of the specific additives. However, such unstable polymorphs would not be suitable drug candidates. Consequently, these trials suggest that STFs can be used to rapidly identify polymorphs suitable for drug marketing by adopting the following strategy: performing a rapid screening test at high supersaturation to crystallize as many forms as possible, followed by an optimization stage to target each polymorph using specific supersaturation ranges and crystallization times. There is a growing library of STFs comprising several different oils,^12–19,46^ and whilst water is usually the other immiscible liquid, with typically either ethanol or propanol as the amphisolvent, non-aqueous STFs have also been reported.^47^ This should enable a suitable STF to be rapidly identified for a particular drug given the key requirement that the crystallizing compound is soluble in one of the immiscible liquids and virtually insoluble in the other. Hence, the method should be generally applicable.

### Comparison with other nm-confined systems

Droplet microemulsions^33,48–50^ and nanoporous systems^51–56^ have been used previously for selective polymorph and allotrope formation. STFs provide significant advantages over both these systems. In particular, crystals grow exceedingly slowly in droplet microemulsions so that the crystals are often limited to sub-μm sizes and, in addition, are significantly contaminated with surfactant that is challenging to remove. In nanoporous systems, the difficulty in extracting sub-μm crystals from the surrounding solid matrix limits applications. In contrast, crystals grow in the STFs at a slowed but reasonable rate, with the choice of either growing crystals to sedimentable dimensions for easy extraction, or restricting the growth to the nm range by using lower initial supersaturations to obtain a nanocrystal suspension. The STF experimental procedure is similar to normal solution crystallization, but with the advantage of a higher nucleation rate and slower growth profile that aids selectivity and early-stage crystallization analysis. Furthermore, the ability to target polymorphs with vastly different stabilities and growth rates from the same formulation is a key benefit.

## Conclusions

In summary, we have revealed that a degree of restricted diffusion in STFs enables crystallization to occur under higher nucleation rate and slower crystal growth regimes that are impossible to access in normal bulk solution crystallization. This restricted diffusion prolongs the lifetime of locally high supersaturations and reduces the rate of Ostwald ripening so that polymorphs that are difficult to nucleate, transitory and/or have slow growth can be targeted. In particular, under relatively low supersaturation conditions, crystallization occurs under thermodynamic control and stable but slow-growing polymorphs, such as γ-glycine, can be obtained. Under higher supersaturation conditions, the even higher local supersaturations generated in STFs lower the nucleation energy barriers to all polymorphic forms, making them more accessible, whilst the restricted diffusion makes them less susceptible to dissolution in the presence of lower energy forms. Hence, metastable polymorphs that are hard to nucleate and often transient, such as β-glycine, can be readily extracted. The greater potential for multiple polymorph discovery within a single STF formulation means that STFs should prove a potent and versatile tool for polymorph discovery, and selective crystallization in general, particularly because they are able to target both stable and metastable polymorphs with vastly different stabilities and crystal growth rates. This could transform the current, scatter-gun approach to polymorph screening, which involves using many different solvents with different cooling profiles to hopefully obtain different polymorphs, but without any guarantee of success.

Furthermore, the STF can act as an array of nanocrystal incubators, with the nanocrystals remaining suspended in the fluid for extended periods of time. Accordingly, aliquots of the nanocrystal containing STF can seed metastable solutions to induce crystallization of the desired polymorph. Here, the longevity of these nanocrystal suspensions, the slow nanocrystal growth so that crystal perfection is maintained, and the ability to target specific polymorphs are key advantages over current seeding capabilities. Finally, the slow, restricted crystal growth rate in STFs should enable unprecedented study of early-stage crystallization to provide new insights. For all these reasons, we hope that this helps initiate a new field of crystallization in STFs.

## Data availability

Experimental methods and experimental supporting data are provided in the ESI.† The datasets supporting this article have been uploaded as part of the ESI material.†

## Author contributions

S. J. C. conceived the idea. J. J. M. performed the investigations. P. H. and J. A. A. advised on the NMR work and draft manuscript and J. A. A. designed the NMR methodology. S. J. C. wrote the original manuscript and all authors contributed to its revision.

## Conflicts of interest

There are no conflicts to declare.

## Supplementary Material

SC-013-D2SC04413G-s001

SC-013-D2SC04413G-s002

SC-013-D2SC04413G-s003

## References

[cit1] Hu Y.-C., Tanaka H. (2022). Nat. Commun..

[cit2] Smith G. D., Donelan C. E., Barden R. E. (1977). J. Colloid Interface Sci..

[cit3] Diat O., Klossek M. L., Touraud D., Deme B., Grillo I., Kunz W., Zemb T. (2013). J. Appl. Crystallogr..

[cit4] Prévost S., Lopian T., Pleines M., Diat O., Zemb T. (2016). J. Appl. Crystallogr..

[cit5] Lopian T., Schöttl S., Prévost S., Pellet-Rostaing S., Horinek D., Kunz W., Zemb T. (2016). ACS Cent. Sci..

[cit6] Zemb T., Klossek M., Marcus J., Schöttl S., Horinek D., Prévost S. F., Touraud D., Diat O., Marčelja S., Kunz W. (2016). Proc. Natl. Acad. Sci. U. S. A..

[cit7] Schöttl S., Lopian T., Prévost S., Touraud D., Grillo I., Diat O., Zemb T., Horinek D. (2019). J. Colloid Interface Sci..

[cit8] Klossek M. L., Touraud D., Zemb T., Kunz W. (2012). ChemPhysChem.

[cit9] Xu J., Zhang L., Yin A., Hou W., Yang Y. (2013). Soft Matter.

[cit10] Xu J., Zhang L., Li C., Zhan T., Hou W. (2013). RSC Adv..

[cit11] Schöttl S., Marcus J., Diat O., Touraud D., Kunz W., Zemb T., Horinek D. (2014). Chem. Sci..

[cit12] Hou W., Xu J. (2016). Curr. Opin. Colloid Interface Sci..

[cit13] Schöttl S., Horinek D. (2016). Curr. Opin. Colloid Interface Sci..

[cit14] EL-Hefnawy M. E. (2012). Mod. Appl. Sci..

[cit15] Xu J., Zhang L., Li D., Zhao J., Hou W. (2013). Colloid Polym. Sci..

[cit16] Xu J., Deng H., Song J., Zhao J., Zhang L., Hou W. (2017). J. Colloid Interface Sci..

[cit17] Jehannin M., Charton S., Corso B., Mohwald H., Riegler H., Zemb T. (2017). Colloid Polym. Sci..

[cit18] Sun B., Chai J., Chai Z., Zhang X., Cui X., Lu J. (2018). J. Colloid Interface Sci..

[cit19] Mirhoseini B. S., Salabat A. (2021). J. Mol. Liq..

[cit20] Subramanian D., Boughter C. T., Klauda J. B., Hammoudac B., Anisimov M. A. (2013). Faraday Discuss..

[cit21] Bučar D.-K., Lancaster R. W., Bernstein J. (2015). Angew. Chem., Int. Ed..

[cit22] Liu Y., Gabriele B., Davey R. J., Cruz-Cabeza A. J. (2020). J. Am. Chem. Soc..

[cit23] Chew J. W., Black S. N., Chow P. S., Tan R. B. H., Carpenter K. J. (2007). CrystEngComm.

[cit24] Dowling R., Davey R. J., Curtis R. A., Han G., Poornachary S. K., Shan Chow P., Tan R. B. H. (2010). Chem. Commun..

[cit25] Little L. J., Sear R. P., Keddie J. L. (2015). Cryst. Growth Des..

[cit26] Towler C. S., Davey R. J., Lancaster R. W., Price C. J. (2004). J. Am. Chem. Soc..

[cit27] Ferrari E. S., Davey R. J., Cross W. I., Gillon A. L., Towler C. S. (2003). Cryst. Growth Des..

[cit28] Dang L., Yang H., Black S., Wei H. (2009). Org. Process Res. Dev..

[cit29] Hwang S., Kärger J. (2019). Magn. Reson. Imag..

[cit30] Moulik S. P., Paul B. K. (1998). Adv. Colloid Interface Sci..

[cit31] Shinoda K., Araki M., Sadaghiani A., Khan A., Lindman B. (1991). J. Phys. Chem..

[cit32] Chernobai G. B., Chesalov Y. A., Burgina E. B., Drebushchak T. N., Boldyreva E. V. (2007). J. Struct. Chem..

[cit33] Chen C., Cook O., Nicholson C. E., Cooper S. J. (2011). Cryst. Growth Des..

[cit34] Weissbuch I., Leisorowitz L., Lahav M. (1994). Adv. Mater..

[cit35] Han G., Thirunahari S., Chow P. S., Tan R. B. H. (2013). CrystEngComm.

[cit36] Han G., Thirunahari S., Chow P. S., Tan R. B. H. (2021). Pharmaceutics.

[cit37] Ramakers L. A. I., McGinty J., Beckmann W., Levilain G., Lee M., Wheatcroft H., Houson L., Sefcik J. (2020). Cryst. Growth Des..

[cit38] Weissbuch I., Torbeev V. Y., Leiserowitz L., Lahav M. (2005). Angew. Chem., Int. Ed..

[cit39] McGinty J. , Yazdanpanah N., Price C., ter Horst J. H. and Sefcik J., in The Handbook of Continuous Crystallization, ed. N. Yazdanpanah and Z. K. Nagy, Royal Society of Chemistry, London, 2020, ch. 1, pp. 1–50

[cit40] Lund G., Holt S. L. (1980). J. Am. Oil Chem. Soc..

[cit41] Stephenson G., Borchardt T., Byrn S., Bowyer J., Bunnell C., Snorek S., Yu L. (1995). J. Pharm. Sci..

[cit42] Yu L., Stephenson G. A., Mitchell C. A., Bunnell C. A., Snorek S. V., Bowyer J. J., Borchardt T. B., Stowell J. G., Byrn S. R. (2000). J. Am. Chem. Soc..

[cit43] Tyler A. R., Ragbirsingh R., McMonagle C. J., Waddell P. G., Heaps S. E., Steed J. W., Thaw P., Hall M. J., Probert M. R. (2020). Chem.

[cit44] Singh A., Lee I., Myerson A. (2009). Cryst. Growth Des..

[cit45] Lévesque A., Maris T., Wuest J. D. (2020). J. Am. Chem. Soc..

[cit46] Liu Y., Xu J., Deng H., Songa J., Hou W. (2018). RSC Adv..

[cit47] Fischer V., Marcus J., Touraud D., Diat O., Kunz W. (2015). J. Colloid Interface Sci..

[cit48] Nicholson C. E., Chen C., Mendis B., Cooper S. J. (2011). Cryst. Growth Des..

[cit49] Hargreaves N. J., Cooper S. J. (2016). Cryst. Growth Des..

[cit50] Buckley P., Hargreaves N., Cooper S. (2018). Commun. Chem..

[cit51] Juramy M., Chevre R., Vioglio P. C., Ziarelli F., Besson E., Gastaldi S., Viel S., Thureau P., Harris K. D. M., Mollica G. (2021). J. Am. Chem. Soc..

[cit52] Bishara H., Berger S. (2019). J. Mater. Sci..

[cit53] Jiang Q., Hu H., Ward M. D. (2013). J. Am. Chem. Soc..

[cit54] Hamilton B. D., Hillmyer M. A., Ward M. D. (2008). Cryst. Growth Des..

[cit55] Ha J. M., Wolf J. H., Hillmyer M. A., Ward M. D. (2004). J. Am. Chem. Soc..

[cit56] Meldrum F. C., O'Shaughnessy C. (2020). Adv. Mater..

