# Supplementary material for: Structured ternary fluids as nanocrystal incubators for enhanced crystallization control
Source: Chem Sci. 2022 Oct 24;13(44):13132–40. doi: 10.1039/d2sc04413g (PMC9667960; doi:10.1039/d2sc04413g)
Supplement: SC-013-D2SC04413G-s003 [file SC-013-D2SC04413G-s003.pdf]

Electronic Supplementary Material (ESI) for Chemical Science.
This journal is © The Royal Society of Chemistry 2022

Electronic Supplementary Information for

Structured ternary fluids as nanocrystal incubators for enhanced crystallization control

Jennifer J. Maunder, Juan A. Aguilar, Paul Hodgkinson
and Sharon J. Cooper

MATERIALS AND METHODS

Materials

The chemicals used were as follows: octan-1-ol (99%, Fisher Scientific), ethanol (≥99.8%, Fisher Scientific), toluene (≥99.8%, Fisher Scientific), isopropyl alcohol (≥99.5%, Fisher Scientific), 5-methyl-2-[(2-nitrophenyl)amino]-3-thiophenecarbonitrile (ROY) (Sigma-Aldrich), α-glycine (≥99%, Sigma-Aldrich) and glycine-1-^{13}C (99 atom% ^{13}C, Sigma-Aldrich). Ultra-high purity water (18.2 MΩ cm) was obtained from a Sartorius arium® comfort water purifier.

Solubility of α-glycine in the STFs

STFs containing ethanol, water and octanol were prepared for the required compositions on a 10 g scale. Excess α-glycine was then weighed and added to each sample vial, which were then sealed using PTFE tape. Each STF composition was made twice, with each duplicate being stored in an oven set at 36°C and 50°C, respectively, for 4 months. After this time, the samples underwent hot filtration, and the residual glycine (which was all still α-glycine) was weighed and its mass recorded. From this, the amount of α-glycine that dissolved, and therefore the solubility of α-glycine in each sample, could be calculated.

Preparation of glycine-containing STFs

STFs containing ethanol, water and octanol at constant ethanol mass ratio of 0.40 were prepared on a 250 ml scale for the lower supersaturation γ-glycine crystallization experiments and on a 25 ml scale for the higher supersaturation β-glycine experiments. The water mass ratio was varied from 0.10 to 0.55, so as to encompass the oil-rich, bicontinuous and water-rich regions, see Fig. 1a.

Typically, the required mass of water and glycine were mixed. Octan-1-ol was then added, followed by ethanol, to achieve the desired composition. These systems were stirred with a magnetic stirrer bar at 323 – 333 K for 1 hour, or until all glycine had dissolved. For crystallizations at supersaturations corresponding to c/c_{sat} of 2.00 or higher, where c_{sat} and c are the α-glycine saturation and actual concentrations, respectively, samples were sonicated in an ultrasound water bath for 2 hours, or until all glycine had dissolved.

Crystallizations in the STFs

Once all the glycine had dissolved, the samples were maintained at an elevated temperature above the saturation temperature for a further hour before cooling to 298 K. Cooling was achieved by either directly placing the samples in a water bath at 298 K (fast-cooling method) or cooling at a controlled rate of 10 K hour^{-1} in an oven (slow-cooling method). γ- and α- glycine crystals were obtained in the STFs using both the fast- and slow-cooling methods, whereas β-glycine crystals were obtained in the STFs using only the fast-cooling method. Crystals were extracted from solution via Büchner filtration after 1 week for α- and γ- glycine and after 10–20 minutes for β-glycine. STF-extracted crystals were washed with the corresponding non-glycine containing STF, and then with ethanol to remove residual octanol. Samples were then left to dry at 333 K.

Seeding experiments

Separate 250 ml STFs were prepared that contained suspended nanocrystals of γ-, α- and β- glycine. For the γ- and α- glycine suspended nanocrystals, STFs containing 0.25 mass fraction of water and

0.40 mass fraction of water, respectively, were used with a relative glycine concentration, c/c_{sat}, of 1.30. These STFs were cooled via the slow-cooling method and were then left at 25 °C for 16 hours for the nanocrystals to form. At this time, the STFs contained 1–5 sedimented crystals of size ≤0.1 mm but the majority of the glycine was still in the STF, either in solution or in the form of suspended nanocrystals.

For the β-glycine suspended nanocrystals, STFs containing 0.25 mass fraction of water at a higher relative glycine concentration, c/c_{sat}, of 2.10 were used alongside the fast-cooling method, with the β-nanocrystals forming within 1 hour at 25 °C, at which point, again, there were 1–5 sedimented crystals present.

For the seeding experiments, a 5.0 ml aliquot of the nanocrystal-containing STF was added to 15.0 ml of a binary solution of 0.40 mass fraction water and 0.60 mass fraction ethanol, which contained glycine at a supersaturation corresponding to c/c_{sat} of 1.30. γ- and α- glycine crystals were extracted for ATR-FTIR analysis from the seeded experiments, and the STFs used for the seeding, after 1 week. β-glycine crystals were extracted for ATR-FTIR analysis from the seeded experiments, and the STFs used for the seeding, after 10–20 minutes, with the shortened timeframe being necessary due to the rapid solution-mediated transformation of β-glycine into α-glycine in unstructured solution.

ROY polymorph screening test

A binary system comprised of 0.475 mass fraction toluene and 0.525 mass fraction isopropanol (IPA), and an STF comprised of 0.100 mass fraction toluene, 0.375 mass fraction water and 0.525 mass fraction IPA, were made on a 20 g scale. ROY was then added to each sample to achieve a relative ROY concentration, c/c_{sat}, of 5.5 at 25°C, with c_{sat} = 30 mg cm^{-3} and 3.5 mg cm^{-3} for the binary system and STF, respectively. The binary and STF samples were sealed with PTFE tape before being heated to 65 °C on a hot plate with stirring until all the ROY was dissolved. Samples were then placed in an oven at 70 °C to ensure full dissolution of ROY. After 1 hour, the samples were removed from the oven, placed in a water bath at 25°C and left to crystallize undisturbed. The samples were carefully monitored and in the first trial, crystals were extracted ≈5-10 minutes after the first appearance of a mass of suspended ROY crystals in the STF. This gave the 4 ROY polymorphs Y, YN, ON and R in the STF. In subsequent tests on the STF system, crystals were extracted at different times to achieve the pure polymorphs. In particular, for the pure Y polymorph, crystals were extracted at earlier times as the Y polymorph grew the fastest (it is both the thermodynamic and kinetic product in the toluene-IPA system). The YN and ON polymorphs appeared next as a mass of suspended needle crystals, and at later times the suspended needle mass sedimented and then disappeared as the R polymorph crystallized. In the binary system, only Y crystals were obtained irrespective of the extraction time, consistent with the Y-polymorph being both the kinetic and thermodynamic product.

ATR-FTIR

Dry crystals were gently ground in a pestle and mortar. ATR-FTIR data was then obtained using a PerkinElmer Spectrum FT-IR spectrometer. A sample was placed on the diamond surface of the main instrument platform before being compressed using a metal anvil. The sample was scanned over a range of 4000 cm^{-1} – 380 cm^{-1}.

Optical microscopy

Optical microscopy data were acquired on an Olympus BX50 microscope equipped with a digital camera. Samples were transferred to a clean glass microscope slide before images were taken. To assess crystal growth rates, standard glass microscope slides were cut in half before spreading silicone grease around the outside to form a border. 2–3 drops of fluid were then transferred to the centre of one of the prepared slides before sealing with the other half of the slide. The sealed sample was then placed on a Linkam heating/cooling block, which was fitted with a central hole to allow light transmission through the sample. The sample was heated to 333 K to ensure all glycine remained dissolved, before cooling to 298 K, where the temperature was held. After the first crystal was identified, images were taken once every minute, with the length and width of the crystal(s) in each sequential image then measured. The increase in circumference per unit time was calculated, and from this, the crystal growth rate was determined.

NMR diffusiometry

NMR diffusiometry techniques allow the estimation of diffusion coefficients by labelling spins spatially, allowing the spins to move, and then reading the label. Diffusion coefficients obtained using these methods can be used to detect restricted diffusion, albeit with the consideration that the boundaries are not rigid, as in the case of classic restricted diffusion studies in solid porous materials.[1] To conduct the investigation, we fixed all the parameters in a $^{13}C\{^{1}H\}$-Doneshot45 pulse sequence, varied the composition of the STF mixture, and calculated the apparent diffusion coefficient.

A 500 MHz Varian spectrometer equipped with a probe able to deliver a nominal maximum pulsed field gradient of 62 G cm^{-1} was used to acquire Diffusion-Ordered SpectroscopY (DOSY) experiments at 298 K. The calibrated maximum gradient strength was 60.95 G cm^{-1}. Standard 5 mm NMR tubes were used. A capillary containing D_2O was used to provide the lock signal.

Oneshot45 spectra[2] were acquired for diffusion measurements. $^{13}C\{^{1}H\}$ diffusion measurements were taken with a diffusion delay (Δ) of 800 ms and diffusion gradient length (δ) of 3 ms. The gradient strength was incremented in 15 steps from 5.58 G cm^{-1} to 46.5 G cm^{-1} in equal steps of gradient squared. For ^{1}H diffusion measurements, the Δ was set to 400 ms and the δ was 2 ms. The gradient strength was incremented in 15 steps from 1.86 G cm^{-1} to 27.9 G cm^{-1} in equal steps of gradient squared. All results were analysed using mono-exponential fittings. To ensure there was no convection, the ^{1}H convection compensated double-stimulated echo DBPPSTE_cc pulse sequence[3,4] was used, with Δ = 200 ms and δ = 2 ms. The diffusion-encoding gradient pulses were set to 27.9 G cm^{-1}. The repetition time was 6.0 s, of which 4.0 s comprised the acquisition time. This was repeated with a diffusion delay of 400 ms. Further setup details are summarized in Table S2. All DOSY spectra were processed using VNMRJ 4.2 software.

Powder X-ray diffraction

To prepare each sample, the glycine crystals were ground gently to make a fine powder to reduce preferred orientation effects, before being spread evenly on a silicon powder specimen holder. Data were collected using a Bruker D8 Advance diffractometer operated at 40 kV and 40 mA. This produced Cu Kα1 and Kα2 radiation at a wavelength of 1.5406 Å. DIFFRAC.SUITE software was used to set up and control each experimental run. The width of the incident X-ray beam was 2, 4, or 6 mm depending on the quantity of the sample. A step size of 0.02° was used to scan 2θ values between 10° and 70°, holding the beam at each angle for 1.00, 1.67, and 2.50 s for the 6, 4 and 2 mm beams, respectively.

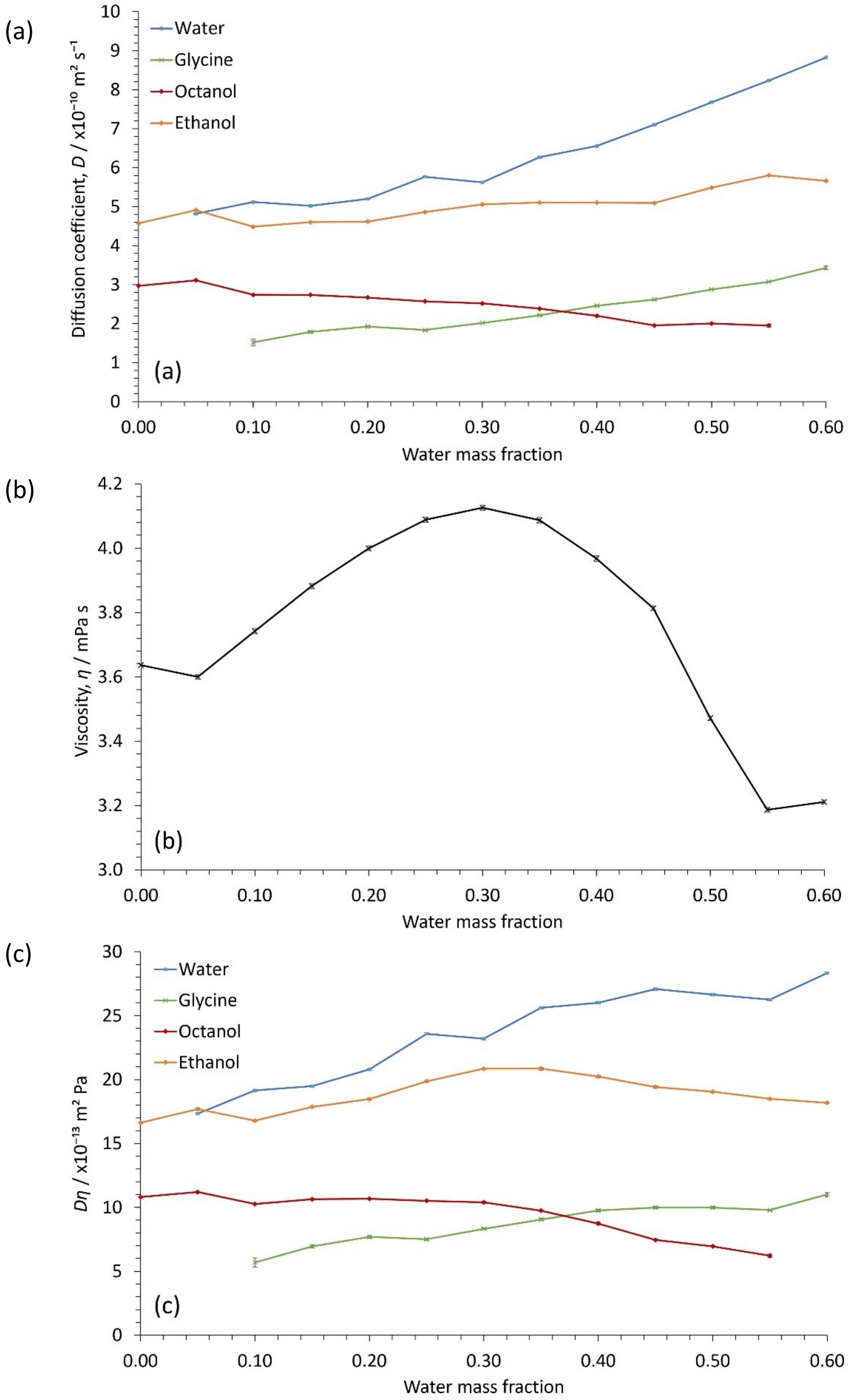

Fig. S1: Degree of restricted diffusion in the glycine containing octanol/ethanol/water mixtures with a fixed ethanol mass fraction of 0.40. (a) Diffusion coefficient values for the STF mixture components, with the glycine-1-^{13}C concentration being 2.14 ×10^{-3} g cm^{-3}, (b) viscosity values of the STF mixtures, and (c) the product $D\eta$ of the STF components. For the viscosity experiments, the glycine amount was varied between 0 and 6 mg cm^{-3}, with this having no effect on the viscosity to within experimental error. The error bars (sometimes concealed by the plot symbols) show the standard error from 3 repeats for each diffusion and viscosity experiment.

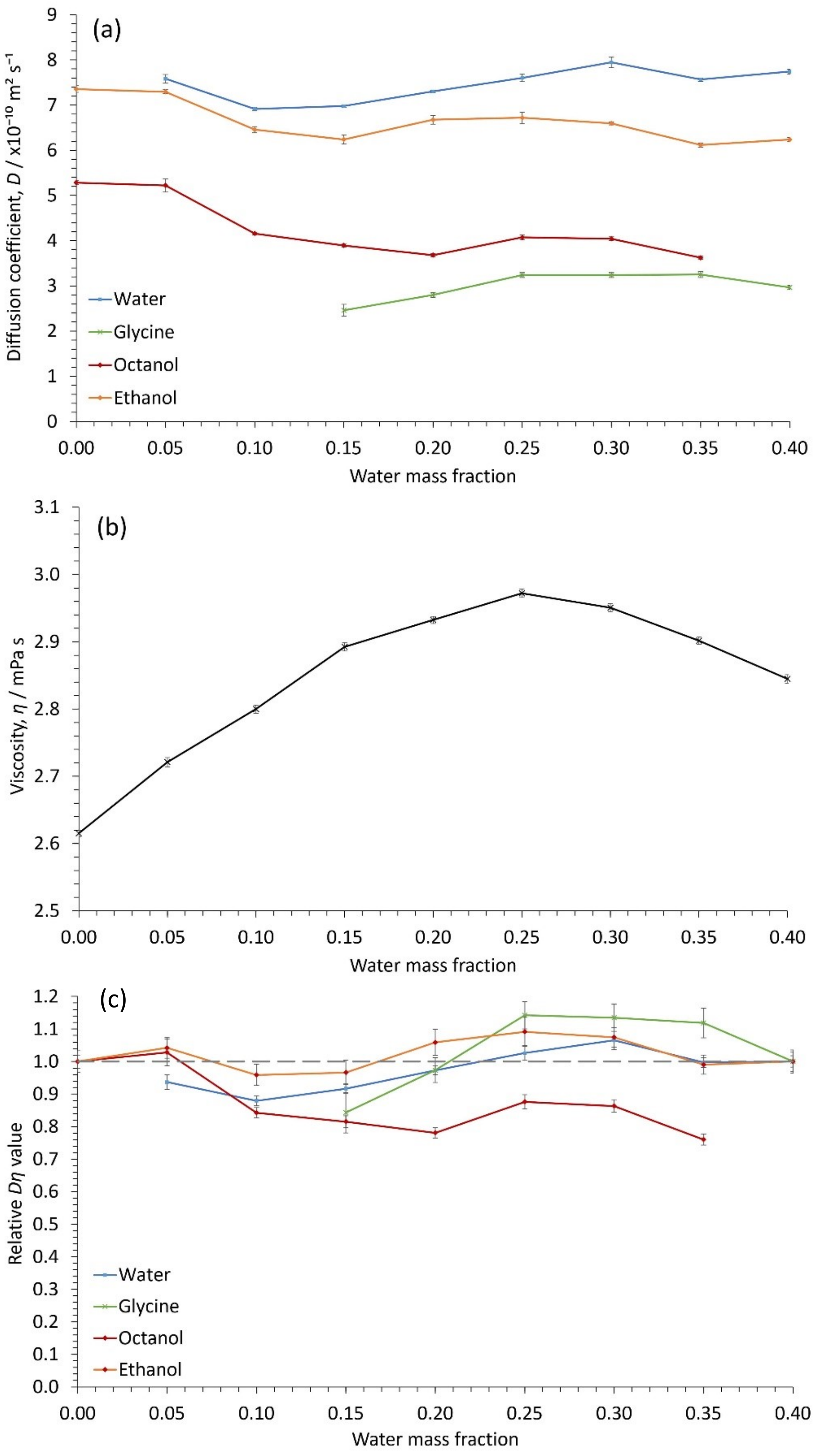

Fig. S2: Absence of restricted diffusion in the glycine containing octanol/ethanol/water mixtures with a fixed ethanol mass fraction of 0.60. a) Diffusion coefficient values for the mixture components, with the glycine-1-^{13}C concentration being 2.14 $\times 10^{-3}$ g cm^{-3} for formulations with water mass fractions of 0.30 and above and saturation values of 0.03, 0.15, 0.21, 0.39, 0.79 and 1.54 g cm^{-3} for formulations with water mass fractions of 0, 0.05, 0.10, 0.15, 0.20 and 0.25, respectively. (b) viscosity values of the mixtures and (c) relative diffusion values obtained from the product Dη relative to the Dη value in the corresponding octanol/ethanol or water/ethanol binary system, i.e. water/ethanol for water and glycine, and octanol/ethanol for octanol. For ethanol, the Dη product is relative to the weighted average of the water/ethanol and octanol/ethanol binary systems The error bars (sometimes concealed by the plot symbols) show the standard error from 3 repeats for each diffusion and viscosity experiment.

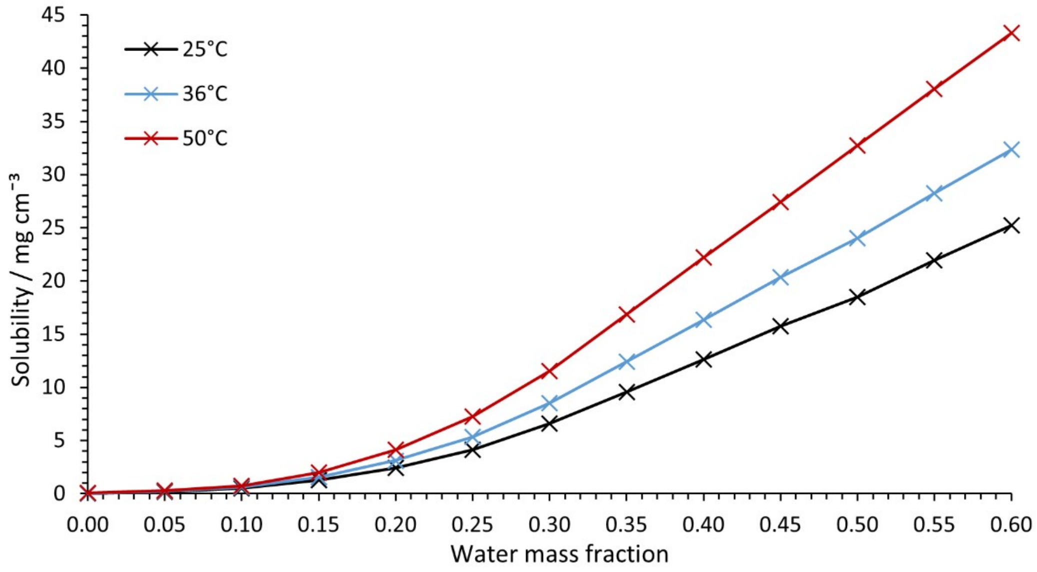

Fig. S3: Solubility of α-glycine after 4 months at 36 ℃ and 50 ℃ in STFs containing a fixed ethanol mass fraction of 0.40. The solubility curve at 25 ℃ was obtained from an extrapolation of the ln(solubility) versus 1/T linear plot.

Table S1: Solubility of α-glycine after 4 months at 36 ℃ and 50 ℃ in STFs containing fixed ethanol mass fractions of 0.4, with the 25 ℃ values obtained from an extrapolation of the ln(solubility) versus 1/T linear plot.

Water mass fraction	Solubility / mg cm^{-3}		
	25°C	36°C	50°C
0.00	0.03	0.04	0.05
0.05	0.18	0.23	0.31
0.10	0.52	0.61	0.74
0.15	1.26	1.56	2.01
0.20	2.45	3.11	4.11
0.25	4.15	5.36	7.22
0.30	6.58	8.51	11.51
0.35	9.56	12.41	16.84
0.40	12.63	16.37	22.19
0.45	15.74	20.32	27.43
0.50	18.47	24.03	32.73
0.55	21.91	28.24	38.03
0.60	25.23	32.35	43.31

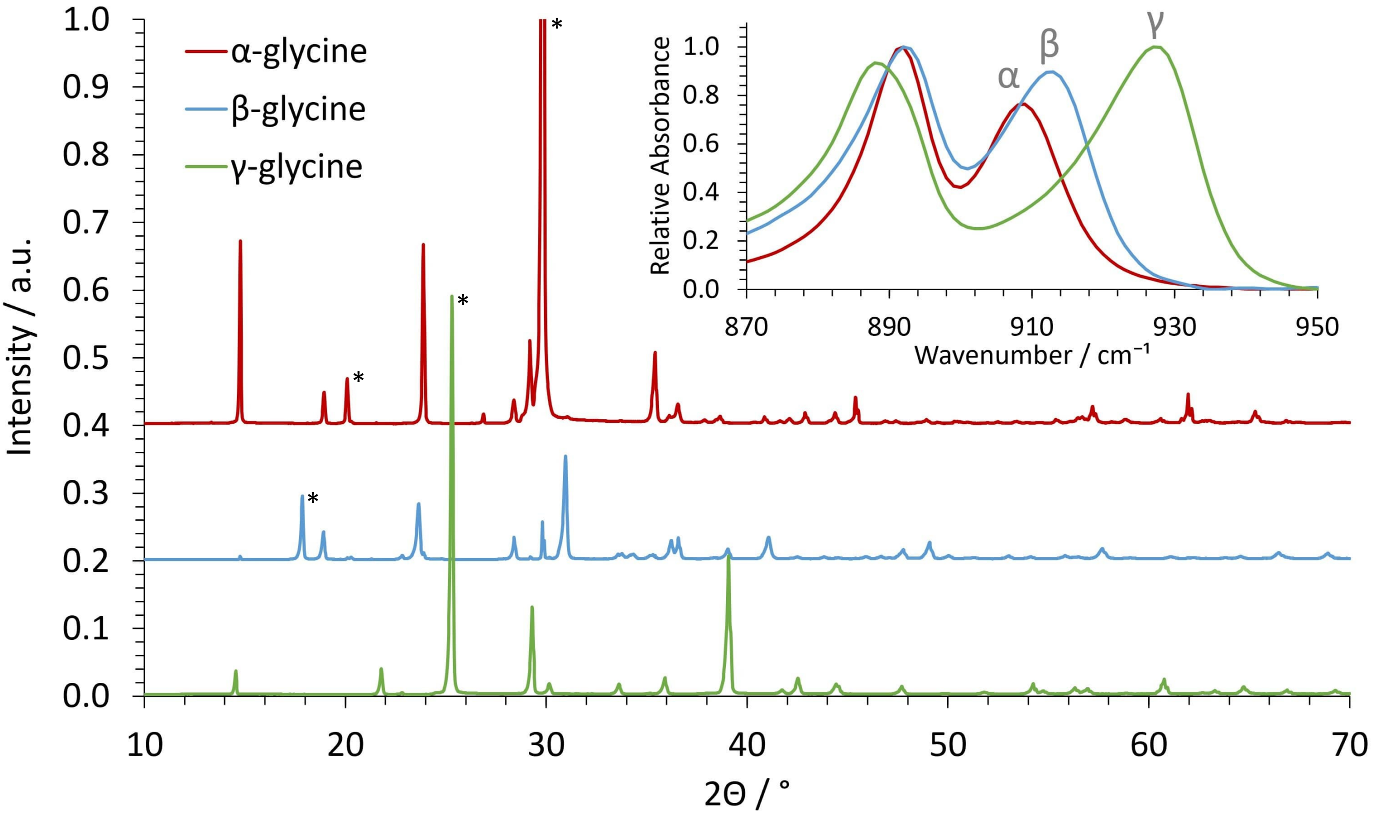

Fig. S4: Representative powder X-ray diffraction (PXRD) spectra of glycine polymorphs obtained from a water-in-oil STF with c/c_{sat} = 1.30 STF (γ-glycine), an oil-in-water STF with c/c_{sat} = 1.30 STF (α-glycine) and a bicontinuous STF with c/c_{sat} = 2.20 STF (β-glycine). The corresponding ATR-FTIR spectra are shown in the inset. The PXRD spectra can be compared to the International Center for Diffraction Data (ICDD) files 00-032-1702, 00-002-0171 and 02-088-4306 for α-, β- and γ-glycine, respectively. Note that characteristic PXRD peaks for α-glycine are at ≈19.5°and 29.7° (asterisked), β-glycine has a characteristic PXRD peak at ≈18° (asterisked) and γ-glycine has a characteristic PXRD peak at ≈25.3° (asterisked).[5-7] The characteristic FTIR peaks for α, β, and γ-glycine at ≈909, 914 and 928 cm^{-1}, respectively.[5,8,9] The β-glycine PXRD spectrum contains trace amounts of the α-glycine polymorph, which can be most easily discerned by the tiny α-glycine peaks at 14.8° and 19.5°.

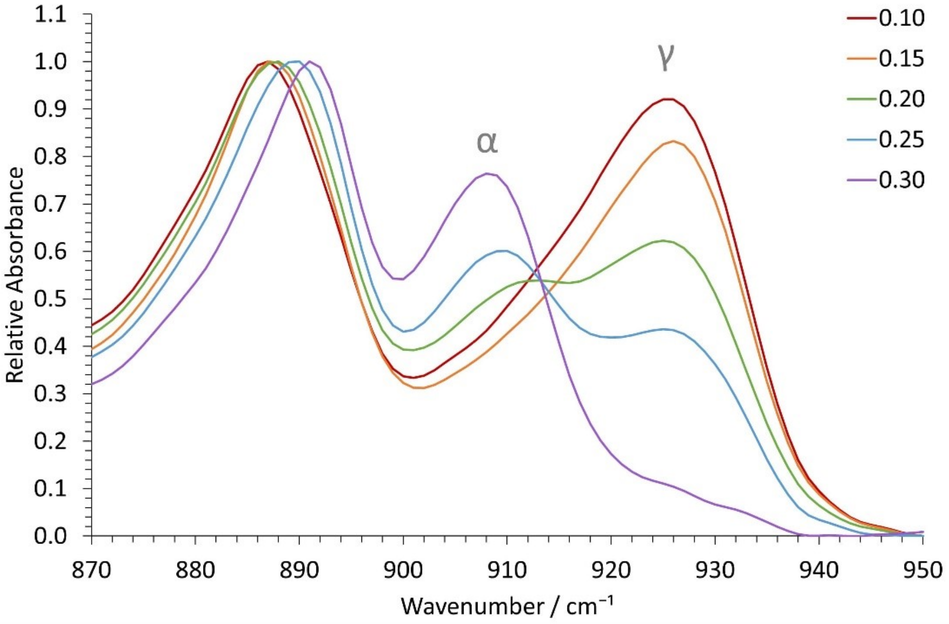

Fig. S5: Representative ATR-FTIR spectra for extracted glycine crystals produced from STFs with varying water mass fractions and a glycine c/c_{sat} = 1.30 using a fast-cooling crystallization methodology. The spectra have been normalized relative to the common peak at 890 cm^{-1}. The γ-glycine and α-glycine peaks are at 928 cm^{-1} and 909 cm^{-1}, respectively.

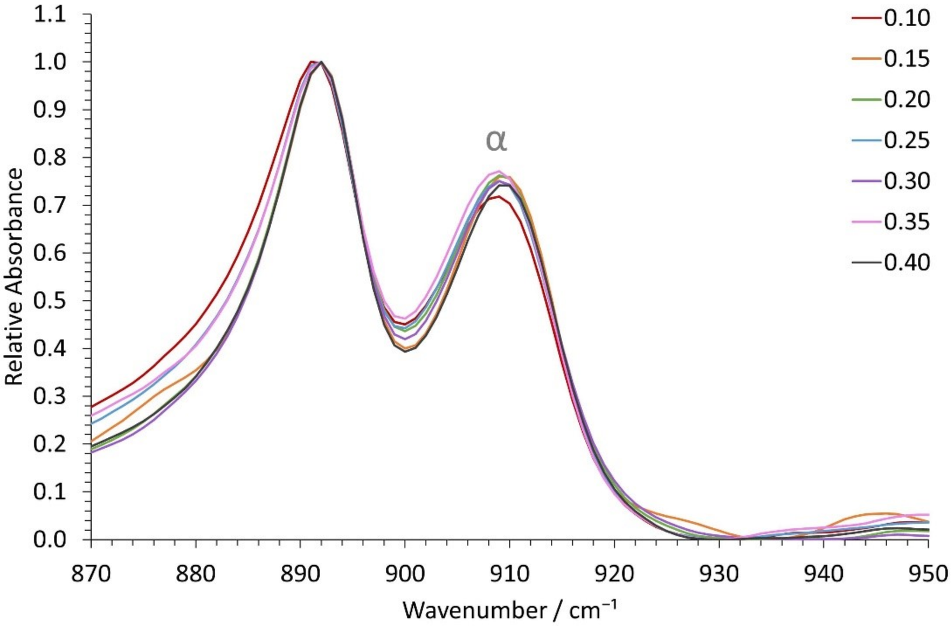

Fig. S6: Representative FTIR data for extracted glycine crystals produced in unstructured octanol/ethanol/water solutions with varying water mass fractions at a fixed ethanol mass fraction of 0.6 and a glycine c/c_{sat} = 1.30. The spectra have been normalized relative to the common peak at 890 cm^{-1}. The α-glycine peak is at 909 cm^{-1}.

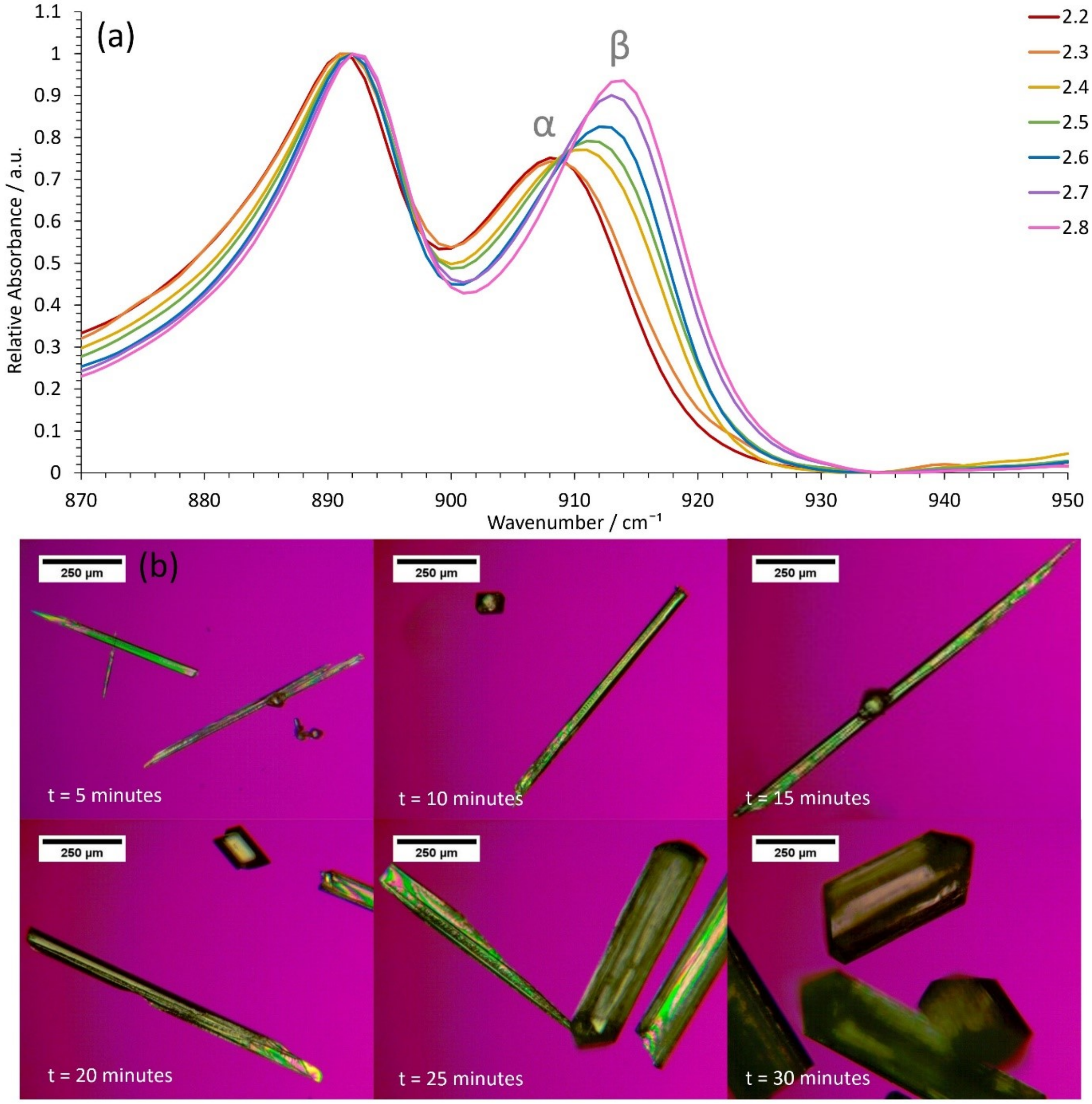

Fig. S7: (a) Representative ATR-FTIR spectra for extracted glycine crystals obtained from binary ethanol and water solutions with ethanol mass fractions of 0.6 and varying supersaturations corresponding to glycine c/c_{sat} of 2.2 to 2.8. The spectra have been normalized relative to the common peak at 890 cm^{-1}. The α-glycine peak is at 909 cm^{-1} and the β- glycine peak is at 914 cm^{-1}. (b) Optical micrographs showing the solution-mediated phase transformation of β-glycine to α-glycine within 30 minutes for the c/c_{sat} = 2.4 system.

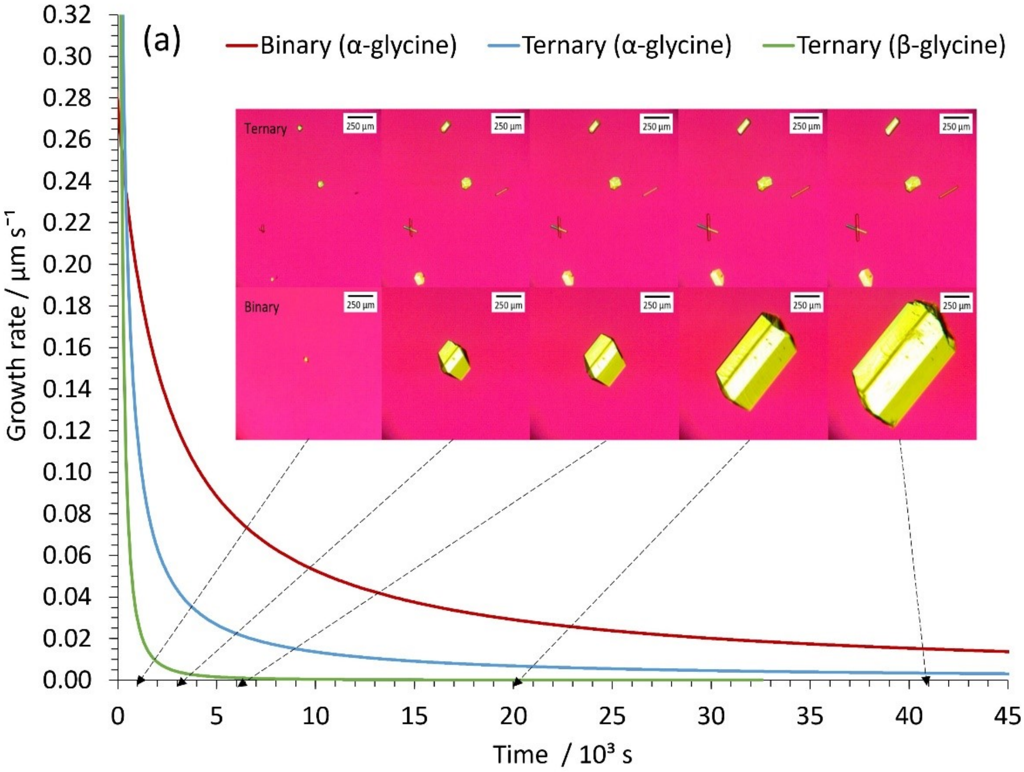

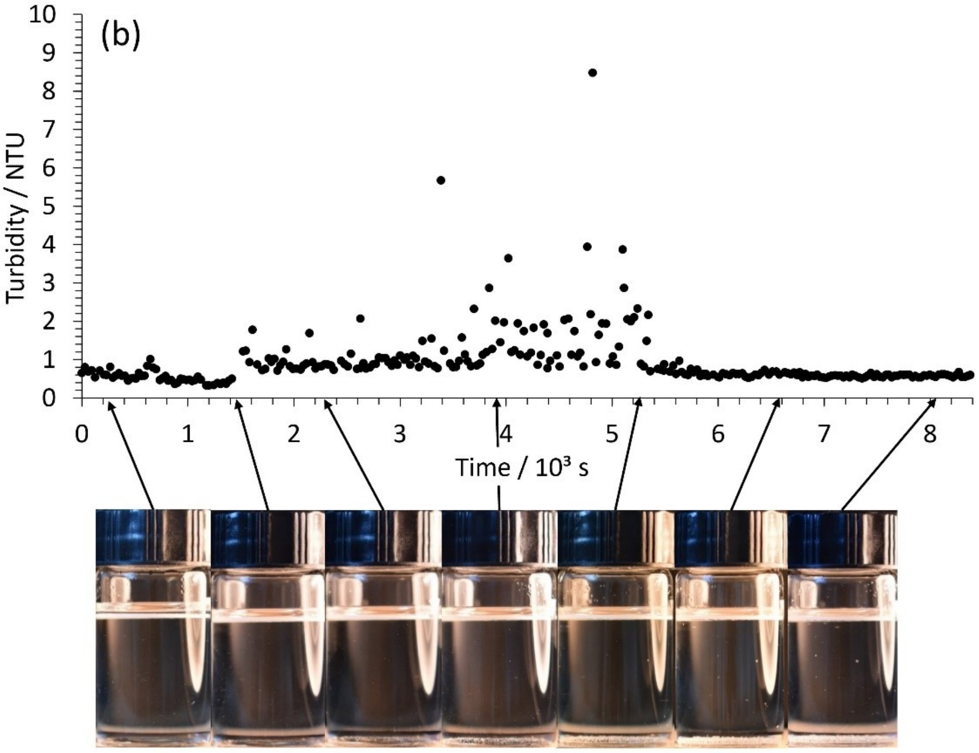

Fig. S8: (a) Comparative growth rate curves for α-glycine in the binary and ternary systems, and β-glcyine in the ternary system, with corresponding optical microscope images. The systems contained an ethanol mass fraction of 0.4 and a glycine c/c_{sat} = 1.90. (b) Turbidity measurements and visual observation of glycine crystallization in the binary system, showing glycine crystals growing at the base of the sample vial and not remaining suspended.

(a)

(b)

(c)

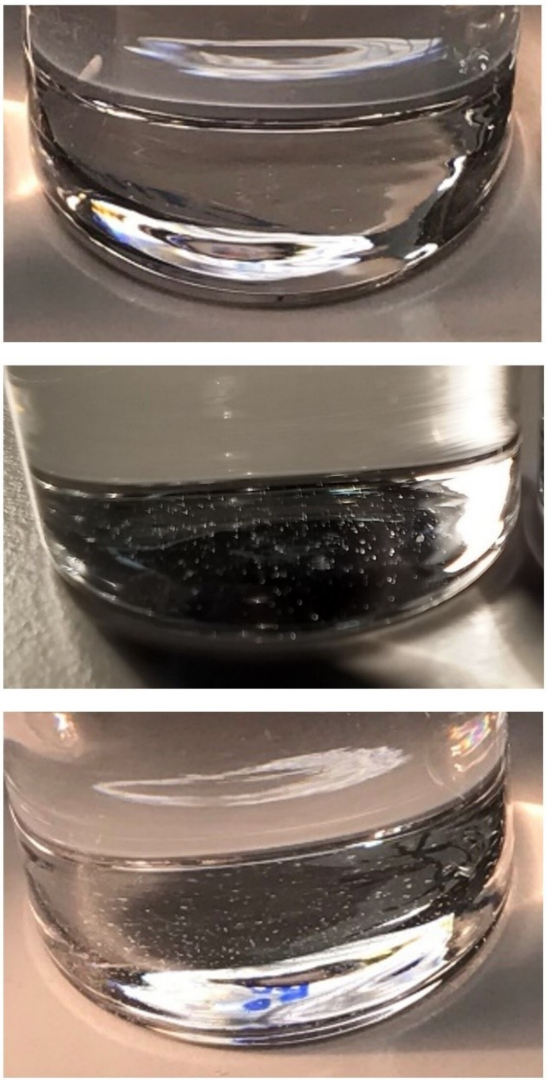

Fig. S9. Visible glycine crystallization in the seeded binary ethanol/water solutions containing 0.60 mass fraction ethanol and c/c_{sat} = 1.30 after (a) 1 hour for γ-glycine crystallization due to seeding with the 0.25 water mass fraction STF with c/c_{sat} = 1.30, (b) 10-20 minutes for β-glycine crystallization due to seeding with the 0.25 water mass fraction STF with c/c_{sat} = 2.10 and (c) 1 hour for α-glycine crystallization due to seeding with the 0.40 water mass fraction STF with c/c_{sat} = 1.30. Note there are far fewer visible crystals in (a) at this stage compared to (c) even though the STF c/c_{sat} = 1.30 value is the same. This is due primarily to the slower growth of γ-glycine compared to α-glycine. In (b), there are more visible crystals than in (a) due to the larger number of seeds in the higher supersaturation c/c_{sat} = 2.10 STF.

Table S2: Summary of the DOSY NMR setup parameters.

Measurement	Calibrated maximum gradient strength / G cm^{-1}	Diffusion delay, Δ / ms	Diffusion gradient length, δ / ms	Spectral width / Hz	Transients	Dummy scans	Complex points	Unbalancing factor, α	Gradient stabilisation delay / ms	Repetition time / s	Acquisition time / s
$^{13}C\{^1H\}$	60.95	800	3	31250	16	4	93750	0.2	2	34	3
^1H	60.95	400	2	8013	32	4	32768	0.2	2	6	4
Convection compensated ^1H	60.95	200, 400	2	8013	32	4	32768	0.15	2	6	4

References

1. W. S. Price, *NMR Studies of Translational Motion. Principles and Applications*, eds. R. Saykally, A. Zewail and D. King, Cambridge University Press, Cambridge, 2009, 120-146.
2. A. Botana, J. A. Aguilar, M. Nilsson and G. A. Morris, *J. Magn. Reson*. 2011, **208**, 270-278.
3. D. Wu, A. Chen and C. S. Johnson, *J. Magn. Reson. Ser. A* 1995, **115**, 260-264.
4. A. Jerschow and N. Müller, *J. Magn. Reson*. 1997, **125**, 372-375.
5. E. S. Ferrari, R. J. Davey, W. I. Cross, A. L. Gillon and C. S. Towler, *Cryst. Growth Des.* 2003, **3**, 53-60.
6. K. Renuka Devi, V. Gnanakamatchi and K. Srinivasan, *J. Cryst. Growth* 2014, **400**, 34-42.
7. G. Han, S. Thirunahari, P. S. Chow and R. B. H. Tan, *Pharmaceutics* 2021, **13**, 262.
8. G. B. Chernobai, Y. A. Chesalov, E. B. Burgina, T. N. Drebushchak and E. V. Boldyreva, *J. Struct. Chem*. 2007, **48**, 332-339.
9. C. Chen, O. Cook, C. E. Nicholson and S. J. Cooper, *Cryst. Growth Des*. 2011, **11**, 2228-2237.
